# Restriction of mitochondrial calcium overload by *mcu* inactivation renders a neuroprotective effect in zebrafish models of Parkinson's disease

**DOI:** 10.1242/bio.044347

**Published:** 2019-09-23

**Authors:** Smijin K. Soman, Michal Bazała, Marcus Keatinge, Oliver Bandmann, Jacek Kuznicki

**Affiliations:** 1 Laboratory of Neurodegeneration, International Institute of Molecular and Cell Biology, Księcia Trojdena 4, 02-109, Warsaw, Poland; 2 Medical Research Council Centre for Developmental and Biomedical Genetics, University of Sheffield, Firth Court, Western Bank, Sheffield S10 2TN, UK; 3 Sheffield Institute for Translational Neuroscience (SITraN), University of Sheffield, 385a Glossop Road, Sheffield, S10 2HQ, UK

**Keywords:** Mitochondria, *mcu*, Parkinson's disease, Zebrafish, CRISPR/Cas9, Neuroprotection

## Abstract

The loss of dopaminergic neurons (DA) is a pathological hallmark of sporadic and familial forms of Parkinson's disease (PD). We have previously shown that inhibiting mitochondrial calcium uniporter (*mcu*) using morpholinos can rescue DA neurons in the *PTEN-induced putative kinase 1* (*pink1*)^−/−^ zebrafish model of PD. In this article, we show results from our studies in *mcu* knockout zebrafish, which was generated using the CRISPR/Cas9 system. Functional assays confirmed impaired mitochondrial calcium influx in *mcu*^−/−^ zebrafish. We also used *in vivo* calcium imaging and fluorescent assays in purified mitochondria to investigate mitochondrial calcium dynamics in a *pink1*^−/−^ zebrafish model of PD. Mitochondrial morphology was evaluated in DA neurons and muscle fibers using immunolabeling and transgenic lines, respectively. We observed diminished mitochondrial area in DA neurons of *pink1*^−/−^ zebrafish, while deletion of *mcu* restored mitochondrial area. In contrast, the mitochondrial volume in muscle fibers was not restored after inactivation of *mcu* in *pink1*^−/−^ zebrafish. Mitochondrial calcium overload coupled with depolarization of mitochondrial membrane potential leads to mitochondrial dysfunction in the *pink1*^−/−^ zebrafish model of PD. We used *in situ* hybridization and immunohistochemical labeling of DA neurons to evaluate the effect of *mcu* deletion on DA neuronal clusters in the ventral telencephalon of zebrafish brain. We show that DA neurons are rescued after deletion of *mcu* in *pink1*^−/−^ and the 1-methyl-4-phenyl-1,2,3,6-tetrahydropyridine (MPTP) zebrafish model of PD. Thus, inactivation of *mcu* is protective in both genetic and chemical models of PD. Our data reveal that regulating *mcu* function could be an effective therapeutic target in PD pathology.

## Abstract (precis)

**Summary:** Mitochondrial calcium overload causes mitochondrial dysfunction in Parkinson's disease. Inactivation of mitochondrial calcium uniporter reverses mitochondrial calcium overload and rescues dopaminergic neurons in our zebrafish models of Parkinson's disease.

## INTRODUCTION

Parkinson's disease (PD) is the second most common neurodegenerative disease affecting 1% of the population above 60 years of age (Kalia and Lang, 2016). The classical pathology includes progressive loss of dopaminergic (DA) neurons in the substantia nigra pars compacta (SNpc), resulting in reduced dopamine levels in the striatum, leading to motor and non-motor symptoms. Unlike sporadic forms of PD which account for 95% of PD cases, familial forms of PD are mainly caused by mutations in *synuclein alpha* (*SNCA*), *parkin*, *PTEN-induced putative kinase 1* (*PINK1*), *DJ-1* and *leucine-rich repeat kinase 2* (*LRRK2*) (Klein and Westenberger, 2012). Many of the gene mutations leading to familial forms of PD in humans are associated with mitochondrial dysfunction (Exner et al., 2012). *PINK1* is a serine-threonine kinase and is an activator of Parkin-mediated ubiquitylation; this quality control process maintains a healthy pool of mitochondria in the cellular system (Valente et al., 2004; Kondapalli et al., 2012).

The mechanism behind the selective loss of DA neurons in the SNpc of PD patients is still poorly understood. DA neurons in SNpc are distinct, as they are autonomously active and employ L-type calcium (Ca^2+^) channels instead of conventional sodium channels to maintain the pace-making activity (Guzman et al., 2015). The pace-making activity of the DA neurons makes the mitochondria susceptible to Ca^2+^ overload. Shortening and simplification of the dendritic arbor observed in neurodegenerative diseases occur through a process of excitatory mitochondrial toxicity, which triggers mitophagy and pre-synaptic mitochondrial depletion, mechanisms that are distinct from classic excitotoxicity (Dagda et al., 2011; Verma et al., 2018). Mitochondrial Ca^2+^ overload leads to neuronal loss in neurodegenerative diseases and ischemia models (Kruman and Mattson, 1999; Kostic et al., 2015; Matthew et al., 2018). The post-mortem tissue samples of the SNpc from sporadic PD patients have a substantial decrease in complex I activity, asserting the role of mitochondrial dysfunction in PD pathology (Schapira et al., 1990). Excessive Ca^2+^ release from endoplasmic reticulum (ER) causes persistent mitochondrial Ca^2+^ overload leading to mitochondrial dysfunction that triggers apoptosis cascade in DA neurons (Lee et al., 2018). Ca^2+^ is transported into mitochondria through mitochondrial calcium uniporter (*mcu*), and mitochondrial Na/Ca^2+^ exchanger (NCLX) is responsible for moving Ca^2+^ out of mitochondria (Baughman et al., 2011). Impaired NCLX function has been implicated to be responsible for mitochondrial Ca^2+^ overload during *pink1* deficiency (Gandhi et al., 2009; Kostic et al., 2015). However, further studies are required to evaluate the contribution of mitochondrial Ca^2+^ influx channels and their regulation in mitochondrial calcium overload. *mcu* is part of a multi-subunit Ca^2+^ channel capable of several states of *mcu* activity. *mcu* is a 40 kDa protein that consists of two coiled-coil domains, two transmembrane domains and a short motif of amino acids between the two transmembrane domains critical for Ca^2+^ transport. Overexpression of *mcu* increases the rate of mitochondrial Ca^2+^ influx and mutation in the highly conserved DIME motif ablates *mcu* activity (Baughman et al., 2011; Stefani et al., 2013).

Zebrafish are an ideal model for studying human diseases, as it is a vertebrate with high fecundity and short generation times. Additionally, drugs can be administered to zebrafish embryos through the aqueous environment. PD is the most studied movement disorder in zebrafish (Vaz et al., 2018). Zebrafish have a well-characterized dopaminergic neuronal system and contain orthologs for approximately 82% of all human disease genes (Howe et al., 2013). The *pink1^−/−^* zebrafish model of PD exhibits classical pathologies seen in human PD cases, such as loss of DA neurons and complex 1 inhibition (Flinn et al., 2013). We have previously reported that inhibition of *mcu* using morpholino and ruthenium red rescues DA neurons in the *pink1^−/−^* zebrafish model of PD (Soman et al., 2017). In this study, we generated an *mcu* null zebrafish line to further determine the underlying mechanisms of the observed rescue effect and in particular, whether DA neuronal loss can be rescued in PD models of zebrafish by eliminating mitochondrial Ca^2+^ overload. In addition to the *pink1^−/−^* zebrafish model of PD, we also used 1-methyl-4-phenyl-1,2,3,6-tetrahydropyridine (MPTP)-treated zebrafish embryos as a model of PD. MPTP is a drug that is often used to induce PD by inducing specific loss of dopaminergic neurons, a decrease of dopamine and motility impairments in zebrafish embryos (Anichtchik et al., 2004).

## RESULTS

### Generation of *mcu* knockout zebrafish using the CRISPR/Cas9 system

**Fig. 1.**
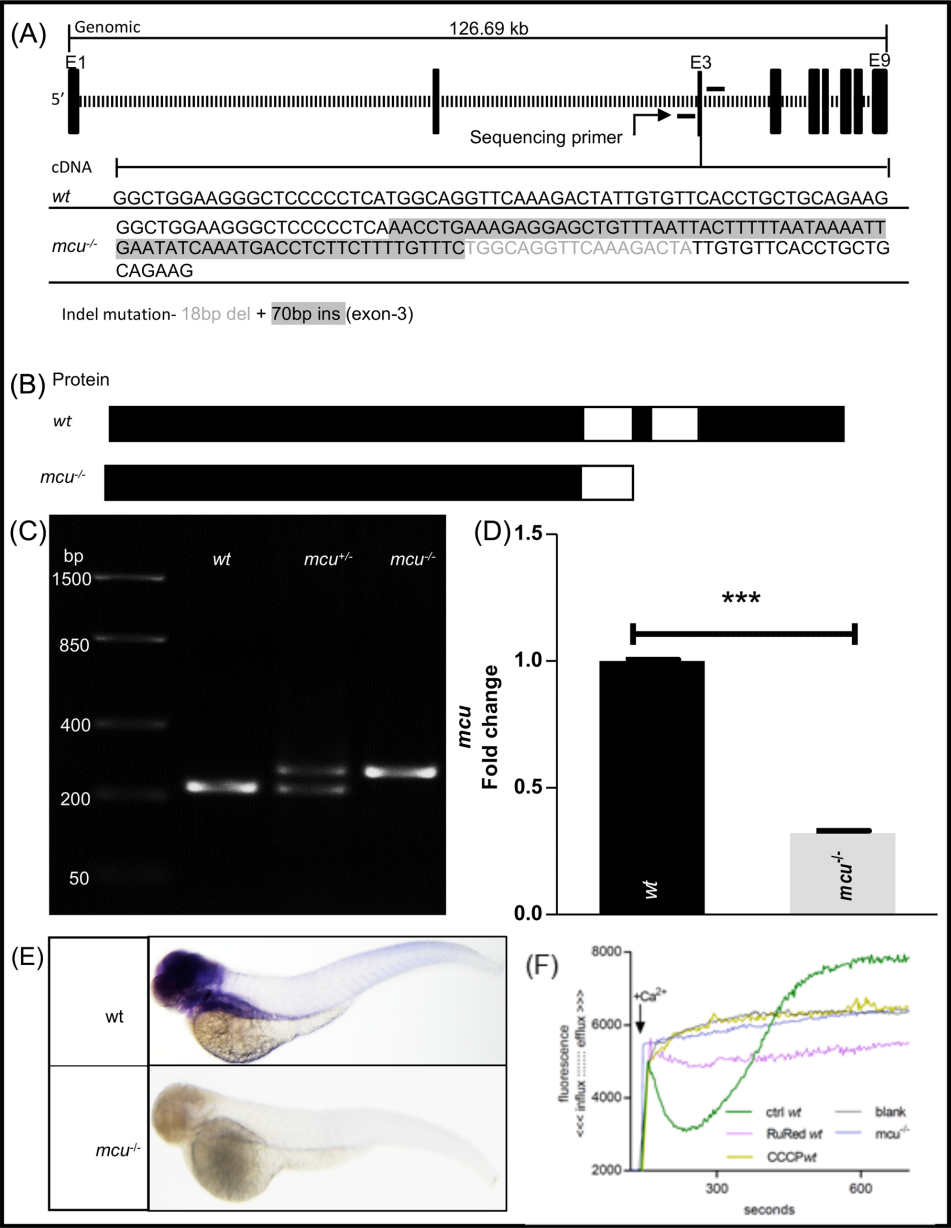
**Generation, screening and validation of *mcu*^−/−^ zebrafish.** (A) CRISPR/Cas9-based gene editing was used to generate *mcu*^−/−^ zebrafish. Schematics showing the targeted genomic sequence for the introduction of the indel mutation in the exon 3 containing the *mcu* coding gene. The identified 18 base pair deletion (shown in faded font) and 70 bp insertion (shown in highlighted font) is denoted in the cDNA sequence. (B) The indel mutation leads to a frameshift mutation with a premature stop codon. The predicted protein product for *mcu* mutant allele is shown in the lower panel. (C) RT-PCR data showing wt *mcu* allele in the first column; the second column shows the wt and mutant allele in heterozygous mutants and the third column shows the *mcu* mutant allele in *mcu* homozygous zebrafish. (D) q-PCR data show significant downregulation (***P<0.0001) of *mcu* gene expression in *mcu*^−/−^ zebrafish when compared to wt, indicating mutation-induced RNA decay. Statistical analysis with *t*-test of three different experiments with *n*=3. (E) Representative images showing *in situ* hybridization using *mcu* riboprobe. *mcu* expression is abolished in *mcu*^−/−^ 3 dpf zebrafish. (F) Impaired mitochondrial calcium ions influx/efflux in isolated mitochondria from 24 hpf larvae in *mcu*^−/−^ zebrafish. Control mitochondria (green line) had an uptake in calcium added to solution, which created a drop in the fluorescence. After ∼5 min, we observed calcium leakage (rise in fluorescence). Blank control (black line) was mitochondria without added calcium. Ruthenium Red (pink line) prevented calcium uptake. CCCP (yellow line) caused leakage of calcium, observed as rising fluorescence. Uptake in *mcu*^−/−^ (blue line) was completely blocked in given circumstances, being like the dynamics of Ruthenium Red treatment.

A frameshift mutation into *mcu* was generated by CRISPR/Cas9 mutagenesis targeting exon 3. The resultant allele from the initial founder screen yielded a 70 bp insertion and an 18 bp deletion (position 13:4750214), confirmed by direct sequencing. F1 embryos from the founders were raised and in-crossed to generate heterozygote, homozygote and wild-type (wt) lines. The PCR results from wt, heterozygous and homozygous larvae are shown in Fig. 1C. Compared with wt larvae, heterozygous larvae exhibited double bands at the site of mutation. The indel mutation resulted in a frameshift mutation and a premature stop codon, leading to a putative truncated protein lacking the transmembrane region. The homozygous *mcu* mutant zebrafish (*mcu^sh214^*=*mcu*^−/−^) line was viable, fertile and no abnormalities in morphology, development or swimming behavior were observed. We performed qPCR to assay *mcu* mRNA level. 70% reduction in *mcu* gene expression was observed at 1 day post fertilization (dpf) in *mcu*^−/−^ when compared to the wt (Fig. 1D) zebrafish. Whole-mount *in situ* hybridization (WISH) revealed that *mcu* expression found in brain, head and liver was significantly diminished in 3 dpf *mcu*^−/−^ zebrafish (Fig. 1E).

### Mitochondrial calcium influx is abolished in *mcu^−/−^* zebrafish

Mitochondrial calcium influx was measured in purified mitochondria of *mcu^−/−^* zebrafish for functional confirmation of *mcu* deletion. Mitochondria from wt and *mcu^−/−^* zebrafish were incubated in a low Ca^2+^ buffer with Ca^2+^-sensitive fluorescent probe (Oregon Green). Ca^2+^ was subsequently added to the medium to detect possible Ca^2+^ uptake by mitochondria. A rapid drop in fluorescence was observed as a result of mitochondrial Ca^2+^ uptake by mitochondria purified from wt zebrafish (Fig. 1F, green line). Then, an increase in fluorescence was observed, indicating Ca^2+^ efflux after the opening of mitochondrial permeability transition pore (mPTP). A similar effect was induced by the ionophore carbonyl cyanide 3-chlorophenylhydrazon (CCCP) (yellow line), which caused mitochondrial membrane-potential loss and efflux of calcium. There was no drop in the fluorescence intensity of purified mitochondria from *mcu^−/−^* zebrafish (blue line), indicating a non-functional calcium influx system after deletion of *mcu*. The fluorescence curve of *mcu^−/−^* zebrafish was similar to the one observed in Ruthenium Red-treated wt zebrafish (magenta line). Ruthenium Red blocks mitochondrial Ca^2+^ uptake by inhibition of *mcu*. In both cases, the constant level of fluorescence signal indicated no Ca^2+^ uptake and thereby suggests a loss-of-function mutation in *mcu^−/−^* zebrafish.

### Mitochondrial Ca^2+^ homeostasis is disturbed in *pink1^−/−^* zebrafish

**Fig. 2.**
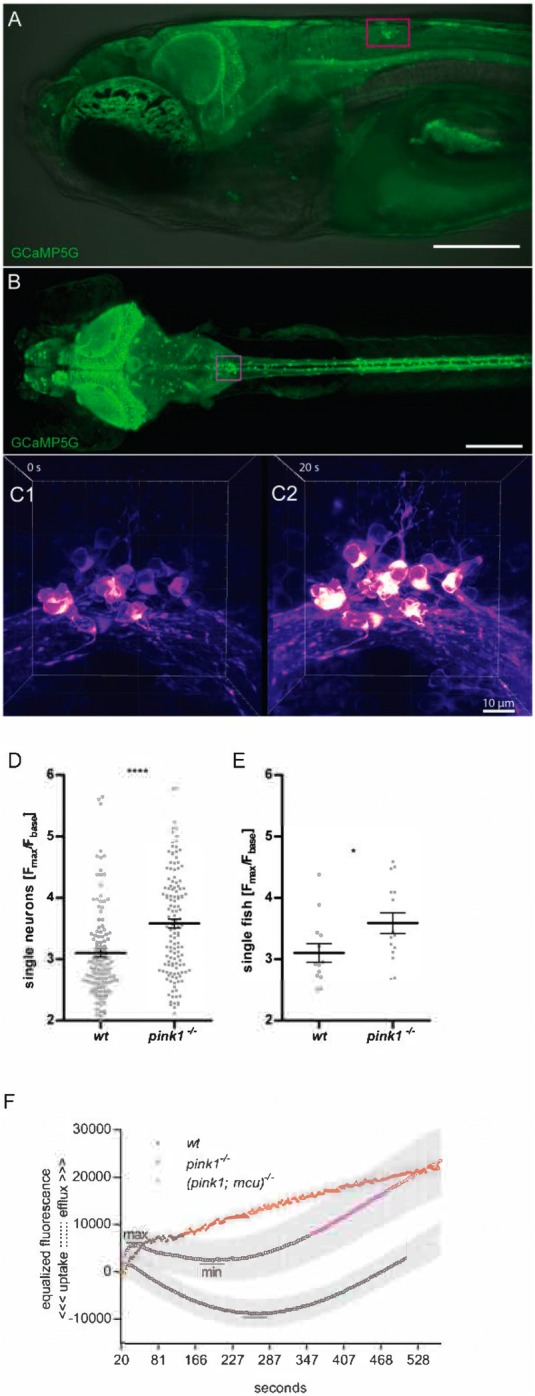
**Mitochondrial calcium homeostasis** **is altered in *pink1*^−/−^ zebrafish.** (A–C) Tg(HuC:GCaMP5G) line with AP localization (A,B) and calcium efflux (C). (A) Lateral view of zebrafish head. Confocal image from GCaMP5G is merged with bright field view image. Scale bar: 100 μm. (B) Dorsal view of AP localization. In A and B, AP is highlighted with a magenta box. Scale bar: 200 μm. (C) The process of calcium efflux in AP neurons before (C1) and after (C2) CCCP treatment. Brighter color represents higher calcium concentration. Scale bar: 10 μm. (D) Fluorescence ratio of calcium released from mitochondria to the cytoplasm to the basal calcium in the cytoplasm, presented as individual single-measured neurons. (E) The same ratios, but presented as individual fish (average from nine neurons). The statistical significance (*P*-value) of two groups of values was calculated using a two-tailed, two-sample unequal variance *t*-test calculated in GraphPad Prism 5. *P<0.05, ****P<0.001. Horizontal bars are means with s.e.m. (wt: *n*=13 fish, *n*=117 neurons; *pink1*^−/−^: *n*=15 fish, *n*=134 neurons). (F) Calcium ions influx/efflux in isolated mitochondria from 24 hpf larvae. On the timeline we can compare the dynamics of calcium influx/efflux in wt, *pink1*^−/−^ and (*pink1; mcu*)^−/−^ mutants. Uptake in *pink1*^−/−^ was lower than in wt, whereas in (*pink1; mcu*)^−/−^was completely blocked in given circumstances. Results are mean with s.e.m. [wt had 11, *pink1^−/−^* had 13, *mcu^−/−^* had three and (*pink1; mcu*)*^−/−^* had four independent replications] gathered during six independent experiments. Every replication was done on 200 larvae per variant.

We analyzed *in vivo* neuronal activity in *pink1^−/−^* zebrafish and Ca^2+^ levels in purified mitochondria. *In vivo* experiments were performed in transgenic zebrafish lines expressing genetically encoded fluorescent Ca^2+^ indicator (GCaMP5G) in neurons. To evoke Ca^2+^ efflux from mitochondrial stores, zebrafish were treated with CCCP. We focused our observations on the area postrema (AP) in the hindbrain region (Fig. 2A,B), since it showed maximum signal strength and encompasses high density of dopaminergic receptors (Tay et al., 2011). As observed *in vivo* by light sheet fluorescence microscopy (LSFM), CCCP induced an increase of GCaMP5G fluorescence in AP neurons of *pink1^−/−^* zebrafish compared to the wt. The increase in fluorescence intensity of the GCaMP5G probe, which is localized in the cytosol, was a result of mitochondrial Ca^2+^ efflux (Brocard et al. 2001) (Fig. 2C1,C2). The ratio of fluorescent intensity at basal conditions (F_min_) to CCCP treatment (F_max_) was calculated and is denoted as F_max_/F_min_. There was a significant increase of F_max_/F_min_ in 3 dpf *pink1^−/−^* zebrafish larvae [3.582 (s.e.m.±0.073; *N*=134)] when compared to 3 dpf wt zebrafish larvae [3.104 (s.e.m.±0.070; *N*=117)] (Fig. 2D). Comparable results were observed when we averaged F_max_/F_min_ from nine measured neurons calculated from every fish (*pink1^−/−^* 3589; s.e.m.±0,17; *N*=15 and wt 3104; s.e.m.±0,15; *N*=13) (Fig. 2E). This indicates that *pink1* deficient condition leads to altered Ca^2+^ homeostasis.

Mitochondrial calcium overload is accounted for in PD pathogenesis, especially in the *pink1*-deficient model of PD (Gandhi et al., 2009; Kostic et al., 2015). Thereby, it was essential to perform a mitochondrial Ca^2+^ influx assay to check whether *pink1^−/−^* zebrafish are also exposed to mitochondrial calcium overload. We observed altered dynamics of Ca^2+^ influx in *pink1^−/−^* compared to wt. Specifically, Ca^2+^ influx was less efficient and lasted relatively shorter in the mitochondria of *pink1^−/−^* zebrafish. This is plotted as a ratio of maximum fluorescence after Ca^2+^ addition (Fig. 2F, max) to the minimum fluorescence during Ca^2+^ influx (Fig. 2F, min). Increased value of the ratio represents efficient mitochondrial Ca^2+^ influx. For wt, it was 1.18 (s.e.m.±0.03; *N*=11) and for *pink1^−/−^*, 1.09 (s.e.m.±0.02; *N*=13) with *P*=0.025, indicating that mitochondrial Ca^2+^ stores in *pink1^−/−^* zebrafish are overloaded.

### *mcu* inactivation enables rescue of dopaminergic neurons in *pink1^−/−^* zebrafish

**Fig. 3.**
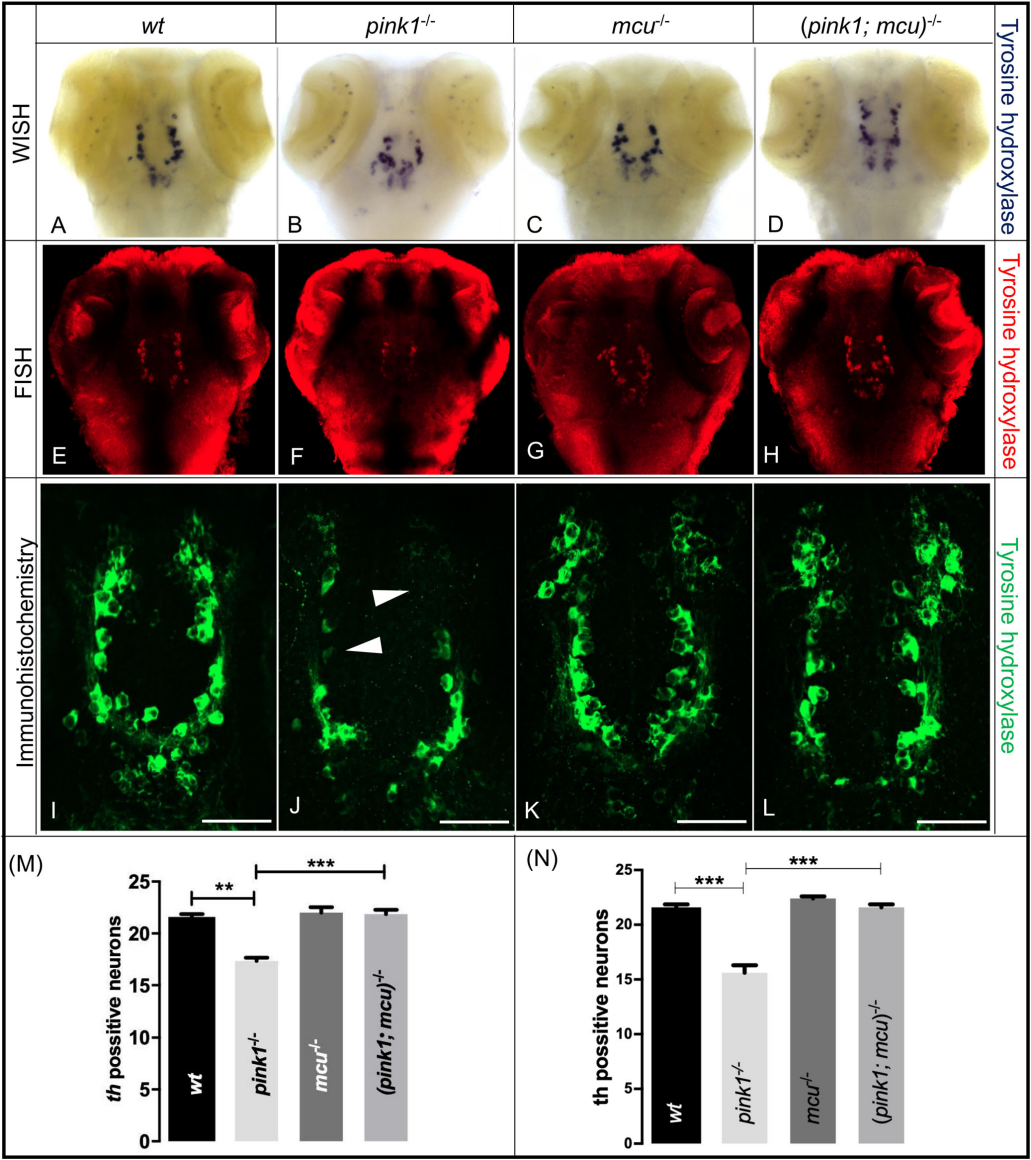
**Dopaminergic neurons are rescued** **after deleting *mcu* in *pink1*^−/−^ zebrafish.** (A–D) Representative images of wt (A), *pink1*^−/−^ (B), *mcu*^−/−^ (C) and (*pink1; mcu*)^−/−^ (D) 3 dpf larvae after WISH using TH-specific riboprobe. (E–F) Representative images of wt (E), *pink1*^−/−^ (F), *mcu*^−/−^ (G) and (*pink1; mcu*)^−/−^ (H) 3 dpf larvae after whole-mount FISH using TH-specific riboprobe and TSA/Cy3-based signal amplification. There was a significant decrease (*P*<0.001) in number of dopaminergic neurons in *pink1*^−/−^ zebrafish when compared to wt. In (*pink1; mcu*)^−/−^ zebrafish there was a significant increase (*P*<0.05) in number of dopaminergic neurons when compared to *pink1*^−/−^ zebrafish. (I–L) Representative images of wt (I), *pink1*^−/−^ (J), *mcu*^−/−^ (K) and (*pink1; mcu*)^−/−^ (L) 3 dpf larvae after immunohistochemistry using TH-specific antibody. Arrowheads show absence of dopaminergic neurons. (M) Graphical representation of chromogenic WISH. There was a significant decrease (**P<0.01) in number of dopaminergic neurons in *pink1*^−/−^ zebrafish when compared to wt. In (*pink1; mcu*)^−/−^ zebrafish, there was a significant increase (***P<0.001) in number of dopaminergic neurons when compared to *pink1*^−/−^ zebrafish. (N) Graphical representation of immunohistochemistry. There was a significant decrease (***P<0.001) in number of dopaminergic neurons in *pink1*^−/−^ zebrafish when compared to wt. In (*pink1; mcu*)^−/−^ zebrafish there was a significant increase (***P<0.001) in number dopaminergic neurons when compared to *pink1*^−/−^ zebrafish. The mean number of diencephalic dopaminergic neurons for wt, *pink1^−/−^*, *mcu^−/−^* and (*pink1; mcu*)*^−/−^* was calculated over three independent experiments (*n*=10 embryos per genotype and experiment). Scale bars: 100 μm.

We next crossed *mcu^−/−^* zebrafish with *pink1^−/−^*zebrafish to generate viable and fertile homozygous double-mutant zebrafish (*pink1*; *mcu*)^−/−^. To analyze the effect of *mcu* inactivation on dopaminergic neuronal viability, WISH and fluorescent *in situ* hybridization using tyrosine hydroxylase (TH) riboprobe were performed in the following experimental groups of 3 dpf zebrafish: wt, *pink1^−/−^*, *mcu^−/−^* and (*pink1; mcu*)*^−/−^* (Fig. 3A–H). Alternative labeling of dopaminergic neurons in the same four groups of fish was performed by immunohistochemistry (IHC) with anti-TH antibody (Fig. 3 I–L). The analysis was focused on TH-labeled neurons concentrated on 1, 2, 4 and 5 subgroups of dopaminergic neurons within the diencephalon (Rink and Wullimann, 2001). It is considered to contain ascending dopaminergic neurons analogous to those in the mammalian SNpc (Rink and Wullimann, 2002). The number of dopaminergic neurons in *pink1^−/−^* 3 dpf zebrafish was reduced by approximately 20% as earlier described (Flinn et al., 2013; Soman et al., 2017) (Fig. 3B,F,J), while in *mcu^−/−^* mutant no loss of dopaminergic neurons was observed (Fig. 3C,G.K). The absence of *mcu* in *pink1^−/−^* 3 dpf zebrafish resulted in the rescue of dopaminergic neurons (Fig. 3D,H,L,). The quantification of labeled neurons showed rescue of DA neurons in (*pink1; mcu*)*^−/−^* double-knockout zebrafish, calculated separately for WISH and IHC (Fig. 3M–N). These results indicate that absence of *mcu* protects dopaminergic neurons during *pink1* deficient condition.

### Mitochondrial membrane potential is partly restored after *mcu* deletion

**Fig. 4.**
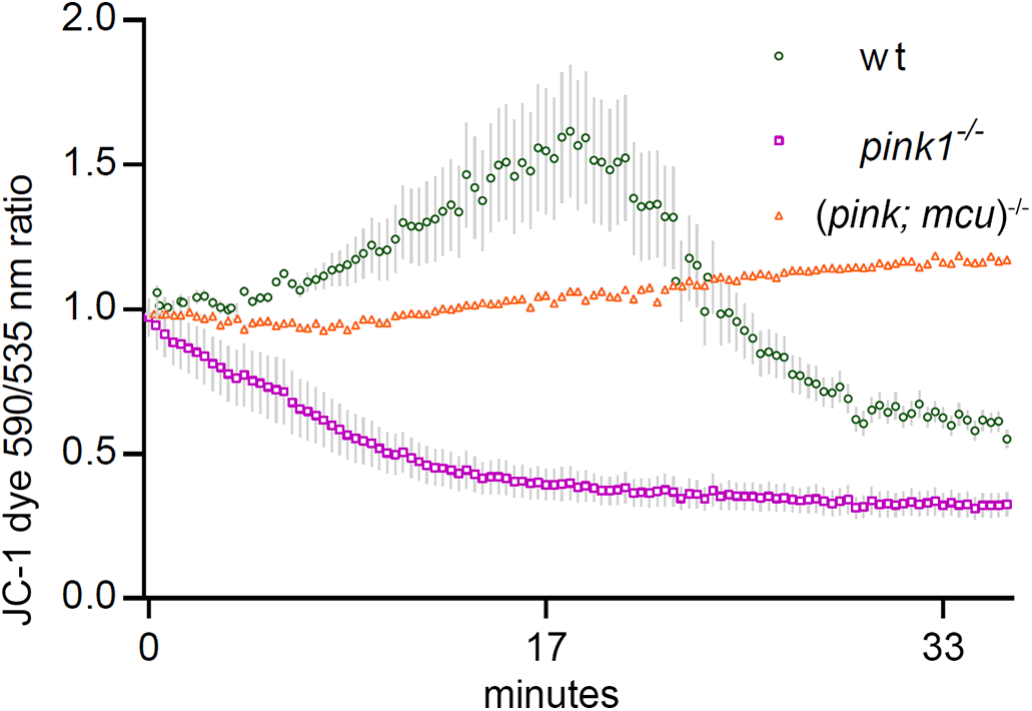
**Mitochondrial membrane potential partly restored after *mcu* deletion.** The graph represents ratio of JC-1 dye emission spectrum (590 nm to 530 nm). wt mitochondria showed a slight uptake of JC-1, which turned into leakage after ∼18 min after the start of the experiment, when mitochondria began to lose membrane integrity in an *in vitro* environment. *pink1^−/−^* mitochondria did not show uptake, instead we observed JC-1 leakage starting at ∼6 min after experiment initialization. Both variants started and ended at similar levels. Results are mean with s.e.m. [wt had two, *pink1^−/−^* had two and (*pink1; mcu*)*^−/−^* had one independent replication(s)] gathered during two independent experiments. Every replication was done on 200 larvae per variant.

Mitochondrial dysfunction is closely associated with PD, and thereby we checked the functional state of mitochondrial membrane potential and mitochondrial respiration. We assayed membrane potential in isolated mitochondria using JC-1 dye. It exhibits membrane potential-dependent accumulation in mitochondria, indicated by the fluorescence emission shift from green (Ex 485 nm/Em 516 nm) to red (Ex 579 nm/Em 599 nm). Mitochondria isolated from wt showed ideal uptake of JC-1 dye and subsequent aggregation leading to a shift in fluorescence from green to red during the first 15 min of measurements. In mitochondria isolated from *pink1^−/−^*, reduced JC-1 aggregation was observed, indicating low mitochondrial membrane potential. Strikingly, in the mitochondria isolated from (*pink1*; *mcu*)*^−/−^* double-mutant, there was an inclination towards JC-1 aggregation, indicating higher mitochondrial membrane potential (Fig. 4).

### Structural alterations in mitochondria

**Fig. 5.**
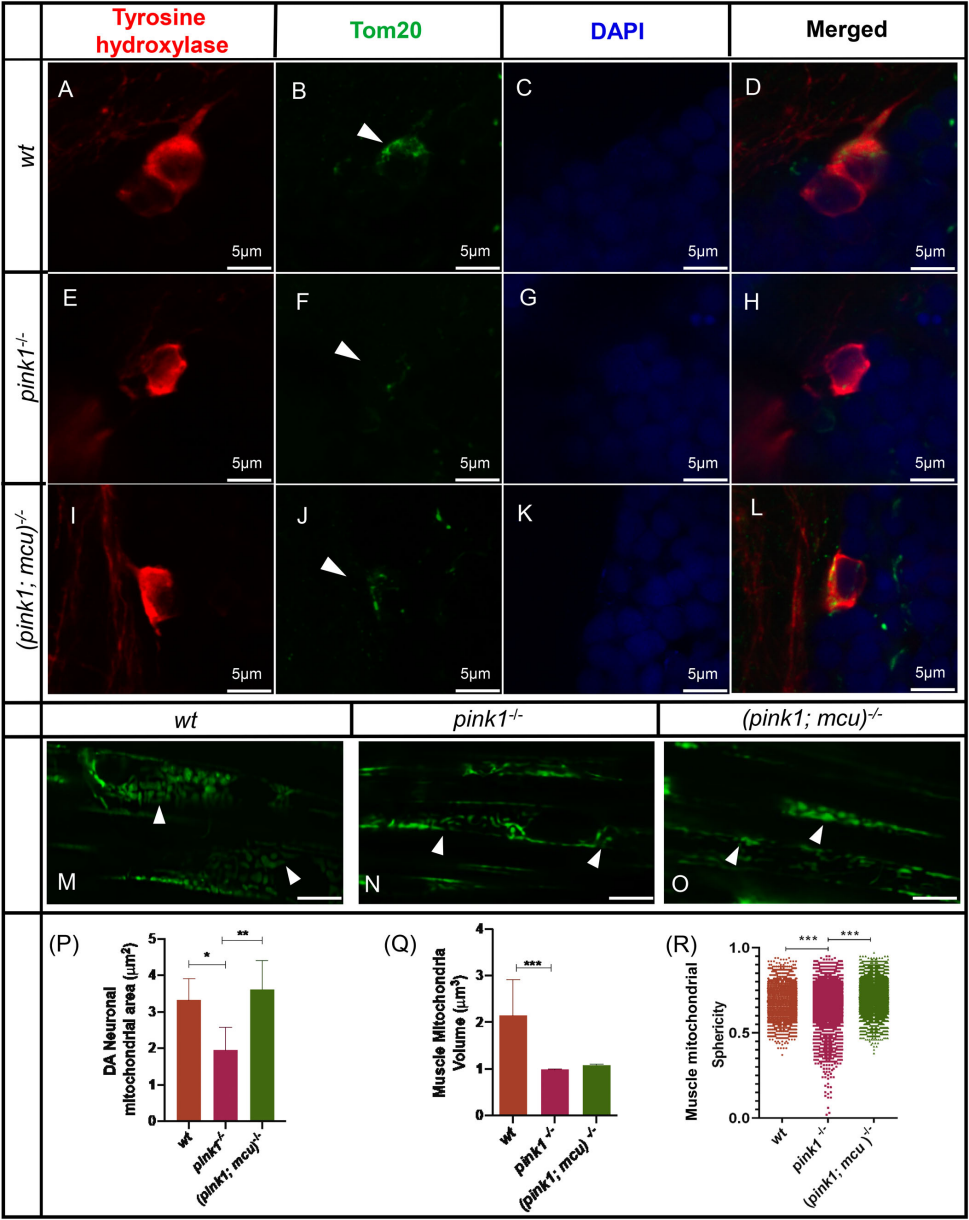
**Mitochondrial dynamics is altered** **in *pink1*^−/−^ zebrafish.** (A–L) Representative images of immunohistochemistry performed on wt, *pink1*^−/−^, *mcu*^−/−^ and (*pink1; mcu*)^−/−^ dissected 4 dpf zebrafish larval brain. DA neurons (red) are marked with anti-TH antibody, mitochondrial structures (green) are marked with anti-Tom20 antibody and DAPI (blue) is shown as nuclear stain. Arrowheads show mitochondrial morphology in muscle fibers. (M–O) Representative images of mitochondrial volumetric analysis in muscle fibers of transgenic wt, *pink1*^−/−^ and (*pink1; mcu*)^−/−^ 3 dpf zebrafish expressing mitochondria-localized GFP. Arrowheads point out mitochondrial structures in somites. (P) Graphical representation and statistical analysis of mitochondrial area with one-way ANOVA and post-hoc analysis using Tukey’s multiple comparison test of two different experiments with *n*=15. There was a significant (*P>0.05) decrease in DA neuronal mitochondrial area in *pink1*^−/−^ zebrafish when compared to wt; mitochondria area was restored (**P>0.01) in (*pink1; mcu*)^−/−^ zebrafish. (Q,R) Graphical representation and statistical analyses of muscle mitochondrial volume and sphericity with one-way ANOVA and post-hoc analysis using Tukey’s multiple comparison test of three different experiments with *n*=30. There was a slight decrease in mitochondrial volume in *pink1*^−/−^ zebrafish when compared to wt. The cumulative sphericity index is significantly (***P>0.001) reduced in *pink1*^−/−^ zebrafish, unlike wt and (*pink1; mcu*)^−/−^ zebrafish.

Mitochondrial dynamics in the form of mitochondrial fission and fusion are a cellular response to mitochondrial stress. In this study, mitochondrial area was investigated in the dopaminergic neurons across the experimental groups. Dopaminergic neurons were marked with TH primary antibody and mitochondrial structures were marked with Tom20 antibody; DAPI staining was added for contrasting background (Fig. 5A–L). There was a significant reduction in mitochondrial area observed in *pink1^−/^*^−^ zebrafish (Fig. 5F,P) when compared to wt (Fig. 5B,P) and *mcu* deletion partially restored mitochondrial area (Fig. 5J,P). Alternatively, mitochondrial volume was studied in muscle fibers using transgenic zebrafish expressing mitochondria-localized GFP [Tg(Xla.Eef1a1:mlsEGFP)] and imaging was performed using LSFM (Fig. 5M–O). There was a significant change of mitochondrial volume in muscle fibers (Fig. 5Q), however *mcu* deletion did not restore mitochondrial volume. Conversely, mitochondrial structure was altered in the form of increased sphericity in *pink1*^−/−^ zebrafish when compared to wt; *mcu* deletion restored mitochondrial sphericity (Fig. 5R).

### *mcu* inactivation lends resistance to MPTP toxicity

**Fig. 6.**
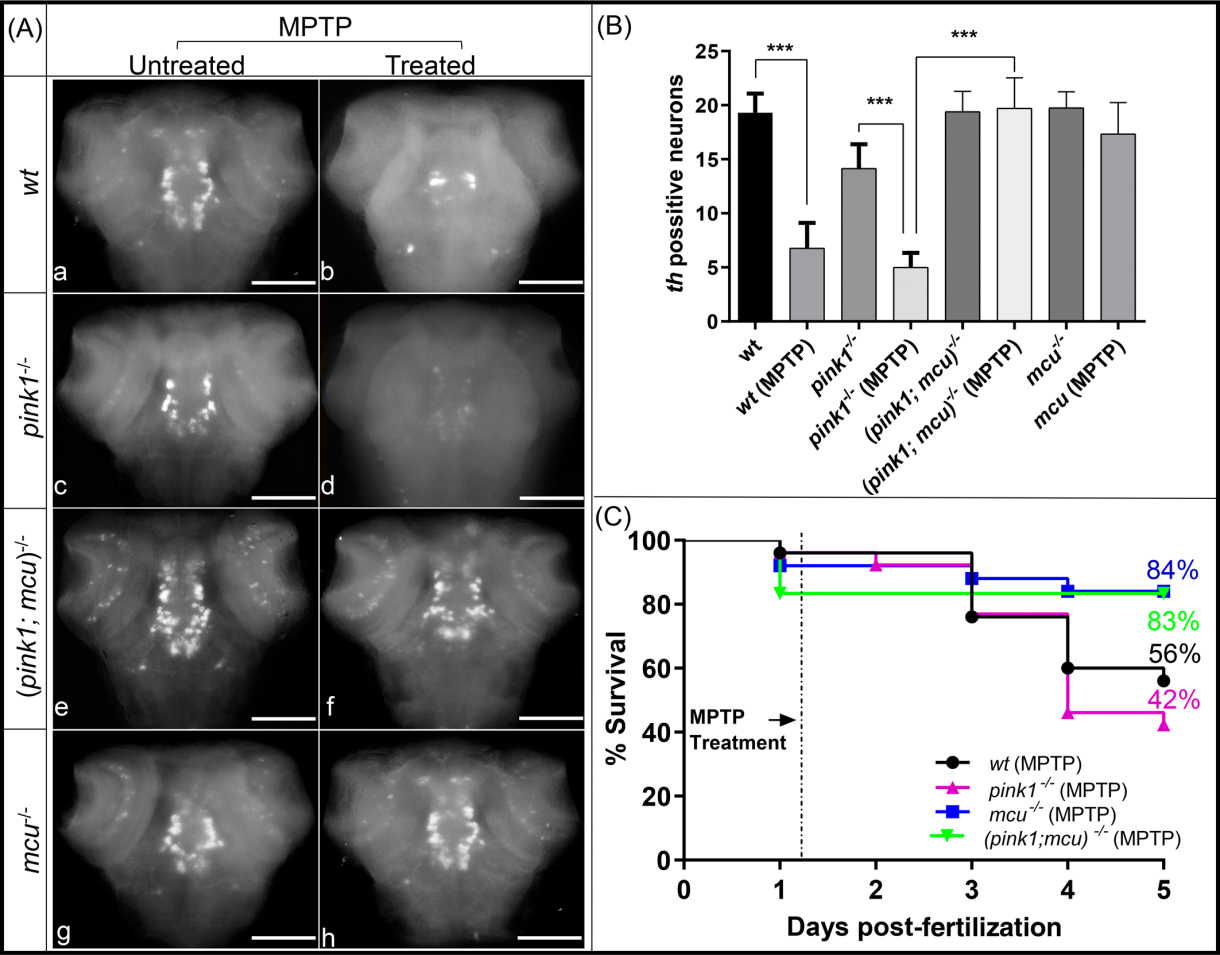
***mcu* inactivation renders neuroprotection against MPTP.** (A) Representative images of MPTP-untreated wt (a), *pink1*^−/−^ (c), (*pink1*; *mcu*)^−/−^ (e), *mcu*^−/−^ (g) and MPTP-treated wt (b), *pink1*^−/−^ (d), (*pink1; mcu*)^−/−^ (f) and *mcu*^−/−^ (h) 3 dpf larvae after WISH using TH-specific riboprobe. MPTP-treated wt (b) and *pink1*^−/−^ (d) zebrafish larvae were most susceptible to MPTP toxicity while MPTP-untreated (*pink1*; *mcu*)^−/−^ (e) and *mcu*^−/−^ (g) were most resistant to MPTP toxicity. (B) Graphical representation of chromogenic WISH. There was a significant decrease (***P<0.001) in number of dopaminergic neurons in *pink1*^−/−^ zebrafish treated with MPTP when compared to untreated *pink1*^−/−^ zebrafish. MPTP-treated (*pink1*; *mcu*)^−/−^ zebrafish showed a significant increase (***P<0.001) in number of dopaminergic neurons when compared to MPTP-treated *pink1*^−/−^ zebrafish. Statistical analysis with one-way anova and post hoc analysis using Tukey's multiple comparison test of two different experiments with *n*=20. (C) Kaplan–Meier survival curves depicting survival rate for 5 days of wt, *pink1*^−/−^, *mcu*^−/−^ and (*pink1*; *mcu*)^−/−^ zebrafish treated with 25 µg ml-1 MPTP (*n*=100). Scale bars:100 µm.

We next checked if lack of *mcu* can protect dopaminergic neurons against toxins such as MPTP, known to induce PD in human and used to generate PD animal models. MPTP is a precursor to MPP+, which is a neurotoxin that acts by inhibiting oxidative phosphorylation causing dopaminergic neuronal death. Following experimental groups of 3 dpf zebrafish wt, *pink1^−/−^*, *mcu^−/−^* and (*pink1; mcu*)*^−/−^* were treated with 25 µM MPTP. 24 h post fertilization (hpf) embryos were monitored for survival after treatment for 5 days and results were plotted as Kaplan–Meier survival curves (Fig. 6C). After treatment with MPTP, there were 56% and 42% survival rates in the wt and *pink1^−/−^* zebrafish, respectively. However, survival of both *mcu^−/−^* and (*pink1; mcu*)*^−/−^* double mutants was much higher; 83% and 84%, respectively. These data show a protective effect of *mcu* deletion on *pink1^−/−^* zebrafish against the Parkinsonian neurotoxin MPTP. Thereby, we investigated the effects of MPTP on the different dopaminergic neural clusters using *in situ* hybridization. The neuronal populations most severely affected by MPTP were the olfactory bulb, pretectal and diencephalic populations. In wt, DA neurons were found to be reduced to a small number of neurons in the locus coeruleus (Fig. 6A), while in the *pink1^−/−^* line treated with MPTP, DA neurons were scarcely present. However, despite MPTP treatment in *mcu^−/−^* and in (*pink1*; *mcu*)*^−/−^* zebrafish, the majority of dopaminergic neurons were present (Fig. 6B). This indicates that lack of *Mcu* prevents the deleterious effects of the neurotoxin on the embryo's dopaminergic system.

## DISCUSSION

We previously reported that *mcu* inhibition using morpholino is protective to dopaminergic neurons in a *pink1^−/−^* zebrafish model of PD (Soman et al., 2017). Thereby, it is imperative to evaluate the neuroprotective role of *mcu* inactivation in a null mutant. In this report, we provide data on the characterization of *mcu^−/−^* zebrafish, generated using a CRISPR/Cas9 approach, and the effects of *mcu* inactivation on mechanisms linked to PD pathogenesis in the *pink1^−/−^* zebrafish model. We show that loss of *mcu* expression results in abated mitochondrial calcium influx in *mcu^−/−^* zebrafish. Our results confirm the role of Mcu in the trafficking of Ca^2+^ into mitochondria of zebrafish, thus providing evidence of *mcu* conservation during evolution. Previous reports have shown that *mcu^−/−^* mice have normal basal metabolism, however under strenuous work conditions *mcu^−/−^* mice succumb to compromised energy production (Pan et al., 2013). Following rodent models and *mcu* morphant zebrafish, *mcu^−/−^* zebrafish are viable, fertile and lack gross morphological aberrations. The ability of vertebrate organisms to thrive irrespective of *mcu* expression is intriguing and could be due to additional Ca^2+^ trafficking channels present in the mitochondria for maintaining basal levels of calcium in the mitochondrial matrix (Hamilton et al., 2018).

There is compelling evidence supporting the role of altered mitochondrial calcium handling in the pathogenesis of PD (Calì et al., 2012; Lieberman et al., 2017; Tabata et al., 2018). Our results in *pink1^−/−^* zebrafish show enhanced CCCP triggered mitochondrial calcium efflux compared to wt zebrafish. Mitochondrial uncoupling by CCCP primes mPTP opening and release of inner mitochondrial calcium stores (Vaur et al., 2000). The increased release of mitochondrial calcium after CCCP treatment indicates mitochondrial calcium overload in *pink1^−/−^* zebrafish. In line with our findings, a recent study showed that GSK3βSer9-mediated blocking of the mPTP leads to increased CCCP-induced mitochondrial calcium release in *pink1* knockdown M17 cells (Parrado-Fernández et al., 2018). *pink1* knockout models have mitochondrial Ca^2+^ accumulation, possibly as a result of mitochondrial Na^+^/Ca^2+^ exchanger dysfunction or altered ER-mitochondria contact sites (Lee et al., 2018). The absence of viable transgenic (*pink1*; *mcu*)*^−/−^* double mutants expressing GCaMP5G redirected us to study calcium influx in purified mitochondria. In a definite trend with our calcium efflux results, extra-mitochondrial calcium influx is increased in purified mitochondria of *pink1^−/−^* zebrafish compared with wt. The double mutants (*pink1*; *mcu*)*^−/−^* showed restored calcium levels. The results from mitochondrial calcium efflux and influx experiments support the notion that mitochondrial calcium overload is prevalent in *pink1* deficiency.

Mitochondrial calcium stimulates adenosine triphosphate (ATP) synthesis, which involves simultaneous generation and salvaging of reactive oxygen species (ROS) (McCormack et al., 1990; Brookes et al., 2004). However, mitochondrial calcium overload can augment ROS generation, leading to inhibition of respiratory chain complex and loss of mitochondrial membrane potential (ΔΨm) (Rizzuto et al., 2012). We had previously shown that inhibiting *mcu* function does not negate mitochondrial respiration and restores complex 1 function in *pink1* deficient condition. We here show that ΔΨm is reduced in purified mitochondria of *pink1^−/−^* zebrafish and *mcu* deletion restores ΔΨm. Previous reports in cell-based models have suggested that a loss-of-function mutation in Pink1 causes a partial decrease in ΔΨm (Abramov et al., 2011; Gandhi et al., 2009). It is intriguing to understand whether decreased ΔΨm is caused by complex I inhibition or oxidative stress during *pink1* deficient condition. In an earlier report, it was shown that loss of ΔΨm could take place independent of mitochondrial respiratory complex inhibitors (Petersén et al., 2000). Thereby it is likely that during *pink1*-deficient condition, altered mitochondrial calcium handling could lead to oxidative stress and thereby loss of ΔΨm.

Mitochondrial structural dynamics are altered as a result of impaired *pink1* mediated mitophagy (Poole et al., 2008; Tsai et al., 2018). Electron microscopy studies in *pink1^−/−^* zebrafish have shown enlarged mitochondrial structures in muscle fibers (Flinn et al., 2013). We see a loss of DA neuronal specific mitochondrial structures in *pink1^−/−^* zebrafish when compared to wt. In contrast, muscle fiber mitochondrial morphology is altered in the form of elongated structures. Previous reports have shown evidence of inter-tissue and intracellular heterogeneity in mitochondrial populations (Kuznetsov and Margreiter, 2009). Additionally, SNpc DA neurons have more reduced mitochondrial volume than SNpc non-DA neurons, making DA neurons more vulnerable to mitochondrial dysfunction (Liang et al., 2007).

The paramount pathology seen in patients with PD is the loss of DA neurons in SNpc. The employment of calcium channels in DA neurons to maintain pace-keeping activity predispose them to mitochondrial calcium overload. Mitochondrial calcium overload is pathological in a diverse array of neurodegenerative diseases including PD and modulation of mitochondrial calcium influx or efflux mechanisms are a plausible strategy for therapeutic intervention in PD.

## MATERIALS AND METHODS

### Animal maintenance

Zebrafish were maintained under standard conditions following international and national ethical and animal welfare guidelines (Local Commission for the Ethics of Animal Experimentation, Warsaw, license number: 657/2015). Mutant lines were generated in the AB background.

### Generation of *mcu^−/−^* zebrafish using the CRISPR/Cas9 system

A DNA ultramer was used to generate the gRNA as previously described (Hruscha et al., 2013). The gRNA was injected into one-cell stage embryos alongside Cas9 protein (NEB). The CRISPR/Cas9 target site contained restriction sites against Mwo1. PCR products prepared from genomic DNA (GenElute™ 96 Well Tissue Genomic DNA Purification Kit, Sigma-Aldrich) extracted from individual embryos at 24 hpf were primarily screened using restriction digestion. Mwo1 sites (GCNNNNNNNNGC) were disrupted in target sites where CRISPR/Cas9-mediated disruption took place. Further screening was done using DNA sequencing. Upon confirmation of indel mutations, the remaining embryos were grown in 28°C till 5 dpf and transferred to aquarium tanks.

### Zebrafish lines

This study was performed in wt AB, *pink1* mutant (*pink1^−/−^*) (Flinn et al., 2013), *mcu* mutant (*mcu^−/−^*) and double mutant for *pink1* and *mcu* (*pink1*; *mcu*)*^−/−^* zebrafish lines. For calcium imaging experiments, the following zebrafish lines were used: Tg(HuC: GCaMP5G) line (Ahrens et al., 2013) and *pink1^−/−^* zebrafish lines were out-crossed, and the 3 dpf embryos were screened for the fluorescent signal. At maturity, Tg(HuC: GCaMP5G^+/−^); *pink1*^+/−^ zebrafish embryos were in-crossed to generate Tg(HuC: GCaMP5G^+/−^); *pink1* (used as a control) and Tg(HuC: GCaMP5G^+/−^); *pink1^−/−^*. The offspring from in-cross of Tg(HuC: GCaMP5G^+/−^); *pink1*^+/+^ and in-cross of Tg(HuC: GCaMP5G^+/−^); *pink1^−/−^* were used for experiments. All zebrafish in the calcium imaging experiments were of mitfa*^−/−^* (nacre) background.

### Chemical treatment

Zebrafish embryos were collected and incubated in E3 medium for 24 h in Petri dishes. After 24 hpf, embryos were segregated into six-well plates with 10 embryos in each. Stock solutions of MPTP (10 mg ml^−1^) (Sigma-Aldrich) were made by adding sterile water directly to the bottle with a needle and syringe. All manipulations with MPTP were performed under a chemical hood. MPTP was diluted in E3 solution to achieve final concentrations of 25 µg ml^−1^. The embryos were kept in an incubator maintained at 28°C. The mortality was noted every day until 5 dpf. On the 5th day, the embryos were washed with fresh E3 solution and fixed in 4% paraformaldehyde (PFA) for 2 h and further processed for *in situ* hybridization.

### RT-PCR

The primers were designed against flanking exons of the target site. RNA was isolated from a pool of 20 embryos using Tri-reagent (Sigma-Aldrich), and cDNA was generated using Verso cDNA synthesis kit (Thermo Fisher Scientific). PCR was performed using a specific primer, using BioMix™ Red (Bioline) master mix.

### qPCR

RNA was extracted from a pool of 20 embryos at 3 dpf using Tri-reagent and cDNA was generated using Verso cDNA synthesis kit (Thermo Fisher Scientific). qPCR analysis was conducted with gene-specific primers (*mcu*: forward, 5′-AGACTGTCAGGAGAGCACAC-3′; reverse, 5′-GACGTACAGAAATCACCGGC-3′), which were optimized for annealing temperature, concentration and efficiency. SYBR Green master mix (Roche) was utilized for the enzyme reaction, which was incubated and analyzed by Light Cycler 96 RT- PCR (Roche). The fold change in expression was quantified by normalizing the threshold cycle (CT) values of the target mRNAs to the CT values of the internal control EF1α in the same samples (ΔCT=CT_target_−CT_EF1α_). It was further normalized with the wt control (ΔΔCT=ΔCT−CT_control_). The fold change in expression was then obtained (2–ΔΔCT).

### Isolation of mitochondria from 24 hpf zebrafish

Mitochondrial isolation from zebrafish embryos was done using the modified method of Prudent (Prudent et al., 2013). 24 hpf embryos were segregated to groups of 200 per variant, washed with Ringer's buffer and dechorionated with pronase solution. Embryos were rewashed with Ringer's until all chorions were removed. Embryos were then deyolked in a loose-fitting homogenizer (Glass-Teflon), filled with 1 ml of 0.5× Ginzburg buffer, with 0.5 mM PMSF and MS-222 (15–50 mg/l) as an anesthetic. After final centrifugation, the pellet was resuspended with 1 ml of mitochondria isolation buffer. Cells were disrupted with a 1 ml syringe and a 26 G×2/3 needle by 50 uptakes. The lysate was centrifuged twice at 1500 ***g*** for 10 min to eliminate nuclei. Then the supernatant was transferred to a pre-chilled 1.5 ml centrifuge tube. It was spun at 10,600 ***g*** for 10 min to pellet mitochondria. Mitochondrial pellet was then suspended in 1 ml of cold KCl medium and centrifuged for 10 min at 10,600 ***g*** at 4°C. Protein amount was established by Bradford protein assay, and samples were equalized to 0.1 µg µl^−1^. Pellet was gently resuspended with 0.15 μl per embryo of KCl medium (125 mM KCl, 2 mM K_2_HPO_4_, 1 mM MgCl_2_ and 20 mM HEPES, pH 7).

### Imaging the AP region

Neurons of AP in the hindbrain region were selected for the experiment and image analysis. They have a relatively large diameter of about 7 μm and convenient localization near the skin (Fig. 2A,B). At 5 dpf, zebrafish were paralyzed by administration of 0.6 µg µl^−1^ pancuronium bromide (P1918, Sigma-Aldrich) in E3 for 7 min (Panier et al., 2013). The immobilized embryo was mounted in the microscopic chamber by submerging in 1.5% low melting-point agarose at 37°C (A9414, Sigma-Aldrich) and drawing the embryos into a glass capillary. Zeiss LSFM Z.1 was used for all imaging experiments. After mounting the zebrafish embryo in the microscopic chamber filled with E3 at 28°C, recording of AP region was initialized, and medium in the chamber was exchanged to E3 with 10 μM CCCP (C2759, Sigma-Aldrich). The acquisition was stopped 1 min after Ca^2+^ efflux. Imaging parameters were as follows; lens Zeiss 40×, 7.5 ms exposure time, 40 z-stack in 39 μm range, ∼1.6 s for one-time lapse. Fish were imaged from the dorsal side of the head and were submerged in the medium from the tip of the head up to the middle of the swimming bladder.

### Measurements of calcium ions uptake/efflux in isolated mitochondria

We compared Ca^2+^ uptake capacity in mitochondria purified from 24 hpf wt, *pink1^−/−^*, *mcu^−/−^* and (*pink1; mcu*)^−/−^ zebrafish. The ability of the assay to detect mitochondrial calcium influx was evaluated using treatment with mitochondrial calcium influx blocker Ruthenium Red and CCCP, which causes mitochondrial calcium efflux due to its mitochondrial uncoupling property. Extramitochondrial-free Ca^2+^ was monitored in the presence of purified mitochondria using Oregon Green 5N hexapotassium salt and the measurement of the fluorescence intensity was performed in the Tecan M1000 fluorimeter (Tecan Trading AG) with parameters: ex/em, 490 nm/525 nm; bandwidth, 15 nm; 20°C. After approximately 20 s of measurement, CaCl_2_ (final concentration=10 µM) was added into wells, supplemented with different reagents based on the variant [0.2 μM CCCP (C2759, Sigma-Aldrich); 10 μM Ruthenium Red (00541, Sigma-Aldrich)]. After addition of reagents, measurements were carried out until fluorescence intensity reached the plateau. Results were plotted onto the graph as an absolute fluorescence level.

### Analyzing mitochondrial membrane potential in isolated mitochondria

We used JC-1 dye (T3168, Thermo Fisher Scientific) in mitochondria isolated from 24 hpf zebrafish. JC-1 is a cationic dye that exhibits potential-dependent accumulation in mitochondria, indicated by a fluorescence emission shift from green (∼525 nm) to red (∼590 nm). We presented results as a ratio of spectra. Isolated mitochondria from 24 hpf zebrafish embryos were transferred to a 96-well non-transparent plate with a flat bottom (Greiner Bio-One), with 80 μl per well (16 μg). Measurement of the fluorescence intensity was performed in the Tecan M1000 fluorimeter (Tecan Trading AG) with excitation 490 nm and emission in two channels: 530 and 590 nm at 20°C. Experiments were conducted until fluorescence showed plateau lines.

### *In situ* hybridization

WISH and fluorescent *in situ* hybridization (FISH) were performed as previously described (Welten et al., 2006; Jowett and Lettice, 1994; Thisse and Thisse, 2008). Briefly, probes for the gene of interest were obtained by PCR using specific primers [*th* (probe length,756 bp): forward 5′AATTAACCCTCACTAAAGGGAGAATGCCGAATTCAAGCAGCTCCAC-3′, reverse 5′-TAATACGACTCACTATAGGGAGAAGCGTGCCGTATGTACTGTGTGC-3; *mcu* (probe length,780 bp): forward 5′-TAATACGACTCACTATAGGGGCTGAGTAAGAAAGCCGAGC-3′, reverse 5′GATTTAGGTACTATAGGCACCACATCCCGAAATCTC-3] and *in vitro* transcription using T7 Polymerase (Roche) with digoxigenin (DIG)-labeled UTP. Embryos were then fixed using 4% paraformaldehyde and stored at −20°C in methanol. After rehydration, permeabilization in 10 µg µl^−1^ Proteinase K, the probe against target mRNA was added to embryos in hybridization buffer (Hyb) at 65°C overnight, then washed by 75% Hyb, 50% Hyb, 25% Hyb in 2x sodium citrate buffer (SSC) and 0.2x SSC. After blocking with 1% bovine serum albumin in the maleic acid buffer, an alkaline phosphatase-conjugated anti-DIG antibody was added and proceeded to react with nitro-blue tetrazolium chloride/5-bromo-4-chloro-3-indolyl-phosphate substrate (Roche, 11681451001). With FISH experiments, anti-DIG-POD (Roche) was used instead. Signal amplification was performed using TSA/Cy3 reagent (Perkin Elmer) according to manufacturer's protocol. Embryos were embedded in 3% methylcellulose and Gelvatol and subsequently, imaging was performed in a bright-field microscope and a fluorescent microscope, respectively. The mean number of these diencephalic dopaminergic neurons for wt, *pink1^−/−^*, *mcu^−/−^* and (*pink1; mcu*)*^−/−^* was calculated over three independent experiments (*n*=10 embryos per genotype and experiment). In order to avoid introducing unintended bias, all embryos were counted with the investigator blinded to the genotype.

### IHC

Whole-mount antibody staining of 5 dpf zebrafish larvae was performed as previously described, with some modifications (Wilson et al., 1990). In brief, embryos were fixed in 4% sucrose/4% PFA at room temperature (RT) for 2 h. Following fixation, embryos were washed in PBS, dissected, dehydrated sequentially in methanol-PBT (PBS+0.5% Triton X-100) and stored in 100% methanol at −20°C at least overnight. Embryos were rehydrated sequentially, washed in PBT, digested with Proteinase K (10 µg µl^−1^ at 120 hpf, for 30 min), and post-fixed in 4% PFA for 20 min at RT. Embryos were blocked for at least 1 h at RT in 10% normal sheep serum/1% DMSO/0.5% Triton X-100 in PBS and incubated in primary antibodies overnight at 4°C. Embryos were washed between four and six times for at least 30 min in PBT at RT and incubated in secondary antibodies for 2 h at RT or overnight at 4°C. Embryos were then washed between four and six times for at least 30 min at RT or overnight at 4°C in PBT and mounted for imaging in prolong gold (Thermo Fisher Scientific, P10144). The following primary antibodies were used: TH [mouse monoclonal IgG1, #ABIN617906, (Antibodies Online, 1:1000)], tom20 [Rabbit monoclonal, #MA5–32148, (Thermo Fisher Scientific, 1:500)]. The following Alexa Flour secondary antibodies were used: Alexa Fluor^®^ 488 [goat anti-mouse IgG (H+L) cross-adsorbed secondary antibody, #A-11001 (Thermo Fisher Scientific, 1:200)], Alexa Fluor^®^ 647 [goat anti-rabbit IgG (H+L) cross-adsorbed secondary antibody, #A-21244 (Thermo Fisher Scientific, 1:200)]. The mean number of these diencephalic dopaminergic neurons for wt, *pink1^−/−^*, *mcu^−/−^* and (*pink1; mcu*)*^−/−^* was calculated over three independent experiments (*n*=10 embryos per genotype and experiment). In order to avoid introducing unintended bias, all embryos were counted with the investigator blinded to the genotype.

### Images post-processing, F_max_/F_min_ factor calculations and data analysis

Time-lapsed images were deconvoluted in ZEN software (Zeiss) using the nearest neighbor method (Castleman, 1979), and converted to Bitplane Imaris 8.3 format. SPOTS function (7 μm estimated diameter, with background subtraction, without region growing option) with object tracking (autoregressive motion) was used in Imaris 8.3 (Bitplane) to automatically find and segregate brightest regions in the 3D region during the time lapse. Specifically, for AP, SPOTS were most frequently found in the large region of the neuron where the axon was attached. This region was rich in the cytoplasm, and thus the fluorescent signal was also high due to the increased level of GCaMP5G localized in the cytoplasm. Region tracking was necessary to overcome muscle contraction movements and drift. Average fluorescent activity plot from every neuron was exported to Microsoft Excel. From the whole region of interest in a single fish, approximately nine neurons were perfectly tracked by the Imaris and those neurons with the highest fluorescent signal in their maximal peak were taken for further calculations and statistics. For each neuron, its average low baseline and its average high peak were calculated from five minimal and five maximal measurements respectively. The mean numbers of high fluorescent peaks and low baselines were calculated from nine neurons per zebrafish. As a determinant of the amount of Ca^2+^ that was released from compartments affected by CCCP, we created the F_max_/F_min_ factor, calculated by dividing the average maximum peak fluorescence by the average minimal baseline fluorescence. We hypothesized that constant efflux of Ca^2+^ from mitochondria triggered by CCCP activity leads to maximum GCaMP5G saturation [K_d_=460 nM (Akerboom et al., 2013)]. In practice, few micromolar Ca^2+^ concentrations saturate GCaMPG5 signal (Badura et al., 2014). Thereby, the longer the saturation lasts, and the higher the F_max_/F_min_ ratio achieved, the more Ca^2+^ was released. The statistical significance (*P*-value) of two groups of values was calculated using a two-tailed, two-sample unequal variance *t*-test calculated in Graph Pad Prism 5. Results are means with s.e.m. (wt: *n*=13 fish, *n*=117 neurons; *pink1^−/−^*: *n*=15 fish, *n*=134 neurons) gathered during five independent experiments.

### Statistical analysis

All the experiments were performed in triplicate unless specifically stated otherwise. Data represent the mean±s.e.m. A minimum of 10 embryos were used per genotype for each replicate experiment. One-way ANOVA and *t*-tests of significance were used unless stated otherwise as measures of significance (Prism, version 7.0; GraphPad Software).
